# Optimized LOWESS normalization parameter selection for DNA microarray data

**DOI:** 10.1186/1471-2105-5-194

**Published:** 2004-12-09

**Authors:** John A Berger, Sampsa Hautaniemi, Anna-Kaarina Järvinen, Henrik Edgren, Sanjit K Mitra, Jaakko Astola

**Affiliations:** 1 Department of Electrical and Computer Engineering, University of California, Santa Barbara, CA 93106-9560, USA; 2 Institute of Signal Processing, Tampere University of Technology, P.O. Box 553, 33101 Tampere, Finland; 3 Biomedicum Biochip Center, University of Helsinki, P.O. Box 63, 00014 Helsinki, Finland; 4 Medical Biotechnology Group, VTT Technical Research Center of Finland and University of Turku, P.O. Box 106, 20521 Turku, Finland

## Abstract

**Background:**

Microarray data normalization is an important step for obtaining data that are reliable and usable for subsequent analysis. One of the most commonly utilized normalization techniques is the locally weighted scatterplot smoothing (LOWESS) algorithm. However, a much overlooked concern with the LOWESS normalization strategy deals with choosing the appropriate parameters. Parameters are usually chosen arbitrarily, which may reduce the efficiency of the normalization and result in non-optimally normalized data. Thus, there is a need to explore LOWESS parameter selection in greater detail.

**Results and discussion:**

In this work, we discuss how to choose parameters for the LOWESS method. Moreover, we present an optimization approach for obtaining the fraction of data points utilized in the local regression and analyze results for local print-tip normalization. The optimization procedure determines the bandwidth parameter for the local regression by minimizing a cost function that represents the mean-squared difference between the LOWESS estimates and the normalization reference level. We demonstrate the utility of the systematic parameter selection using two publicly available data sets. The first data set consists of three self versus self hybridizations, which allow for a quantitative study of the optimization method. The second data set contains a collection of DNA microarray data from a breast cancer study utilizing four breast cancer cell lines. Our results show that different parameter choices for the bandwidth window yield dramatically different calibration results in both studies.

**Conclusions:**

Results derived from the self versus self experiment indicate that the proposed optimization approach is a plausible solution for estimating the LOWESS parameters, while results from the breast cancer experiment show that the optimization procedure is readily applicable to real-life microarray data normalization. In summary, the systematic approach to obtain critical parameters in the LOWESS technique is likely to produce data that optimally meets assumptions made in the data preprocessing step and thereby makes studies utilizing the LOWESS method unambiguous and easier to repeat.

## Background

DNA microarray technology has become a standard tool in biomedical research for large-scale transcriptional monitoring [1]. A growing number of microarray experiments seek to compare samples labeled with two different dyes, such as Cyanine5 (Cy5) and Cyanine3 (Cy3). However, several studies report that the dyes bind on a microarray slide differently due to the variations in their chemical characteristics [2-6]. In addition, the image scanner settings also affect dye intensity measurements. Should these discrepancies not be corrected, the resulting data may not be useful for analysis purposes. Thus, there is a need for dye normalization for the microarray slide prior to actual data analysis to reduce systematic variability.

Microarray data preprocessing contains three phases: quality control, within-slide normalization, and between-slide normalization. Within-slide normalization aims to correct dye incorporation differences which affects all the genes similarly, or genes with the same intensity similarly [7]. One scatterplot-based normalization technique that is particularly suitable for balancing the intensities is called locally weighted scatterplot smoothing (LOWESS) and its original application was for smoothing scatterplots in a weighted, least-squares fashion [8]. This technique is typically chosen to calibrate microarray data because a popular, freely available implementation is available in the statistical software package R [9] and in many commercial microarray analysis software suites such as the Agilent Feature Extraction Software. Moreover, several other freely available microarray data handling packages have incorporated this normalization technique [10,11]. It is noted that many normalization studies simply call the function without rigorous consideration for the actual algorithmic parameters [12,13]. Our analysis reports that the choices of different parameter values drastically affect the quality of the normalization results. The original work on LOWESS clearly mentions the problem of obtaining parameter values and even offers some ideas for finding suitable data-dependent choices [8,14]. However, many microarray studies have omitted such rationale and made arbitrary selections for different experimental data sets [13,15,16] and some studies even failed to report their parameter assumptions in their methods [17-19]. Although this practice has not lead to significant consequences for most of the parameters in LOWESS, we show that the parameter that represents the fraction *f *of neighboring samples to be included in the weighted polynomial fit is particularly sensitive and its variation greatly affects the normalization results. This parameter should be carefully chosen through a systematic procedure where experimental assumptions are clearly specified. Benefits in the normalization process may be considered to be small in their own right, but these improvements are extremely meaningful in the context of searching for subtle biological differences in gene expression.

We outline an optimization-based procedure for obtaining a systematic value for *f *in print-tip LOWESS normalization. Results are compared to common, arbitrary selections of *f*. The proposed procedure first examines a case study where we have utilized three quality filtered, self versus self hybridization experiments. With self versus self experiments, we are able to clearly detect normalization differences. Such analysis also verifies that the optimized method produces properly calibrated ratios. Our proposed technique is also demonstrated on a typical set of quality filtered microarray data. We utilize a set of breast cancer data that has replicated measurements for four different tumor cell lines [20]. In addition to visual comparisons, we quantitatively assess the performance of the different normalization procedures using a goodness-of-fit test. Our results demonstrate that arbitrarily selecting the LOWESS bandwidth parameter produces statistically different results for certain print-tips compared to the proposed optimized parameter selection formulation. Moreover, for genes that have been verified using reverse transcription-polymerase chain reaction (RT-PCR) experiments, we show that calibrated results are substantially affected by the choice of *f*. Our self versus self data, including the original TIFF images, are available online [21] and the replicated breast cancer data is posted by the original authors of that study [22].

## Results and discussion

### Within-slide normalization

Within-slide normalization is used to correct the dye intensity errors introduced across one microarray slide. The result of this step provides the normalized, calibrated ratios. Let 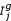 denote the background corrected selection for the intensity of the *j*th gene of the Cy3 (green) colored sample. Similarly, let 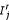 denote the *j*th gene of the Cy5 (red) colored sample. One key issue for the dyes is that they are consistently imbalanced [12,13]. Different labelling effciency between the two fluorescent dyes exists and in some labelling schemes Cy5 is systematically less intense than Cy3. Normalization techniques are needed in order to render the gene expression levels measured by the two different dyes comparable [23,24]. Dye biases can stem from a wide variety of factors, including physical properties of the dyes, effciency of dye incorporation, and processing errors. Such errors may be introduced by slight variations in the amount of mRNA used to create the target hybridized to each microarray or in the quantity of dye used to fluorescently label each target.

For a single microarray experiment, there are *n *total gene expression ratios and we denote the observed vector of ratios for a single experiment as **r **∈ ℝ^*n *× 1^. The calibrated ratio of expression for each gene is obtained by dividing the test by the reference sample intensities with the proper normalization factor in the denominator,


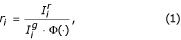


for *i *= 1, 2..., *n*, where *n *is the total number of spots on a microarray. The normalization factor, denoted by Φ(·), is a function of data-dependent variables. If the dyes are linearly dependent, it can be assumed that the normalization function is a constant, namely Φ(·) = *φ*. Many studies have looked at linear dependencies [25], as well as a generalized form of the normalization factor Φ(·) that is a function of an often times unknown number of experiment-specific parameters.

Many studies perform within-slide normalization in a global manner by assuming the error effects are stationary across an entire slide. This is currently true for the cases of Affymetrix GeneChip or Agilent oligonucleotide microarrays. For cDNA microarrays, however, the sources of variation typically originate in a localized or spatial manner [13], mainly from the different print tips for each sub-array of the slide [26]. The process of determining the values for Φ(·) is highly dependent on the characteristics of the data for each print-tip [12]. For example, some print-tips have highly nonlinear effects, while other print-tips in the same experiment behave quite differently and may exhibit linear trends in dye bias. Furthermore, the systematic manner in which the experiment has been conducted also influences the results of different slides, but it is our intention that such effects will be satisfactorily captured in the behavior of the print-tip statistics. As a consequence, we omit global calibration considerations that neglect print-tip distinction and focus solely on scatterplot-based normalization in a termed localized manner.

### LOWESS method

One of the most widely used nonlinear correction techniques is the LOWESS method, which was first applied to microarray data by Yang *et al*. [16]. The main idea behind LOWESS is to utilize a locally weighted polynomial regression of the intensity scatterplot in order to obtain the calibration factor. Compared to other techniques, like housekeeping-based normalization or dye-swap experiments, scatterplot-based normalization is more robust in many types of scenarios where assumptions of constantly expressed genes may break down [23]. Subsequent microarray studies have also chosen this method due to the robustness of fit in the presence of a few extreme outliers. Original studies have examined the (*I*^*g*^, *I*^*r*^)-scatterplot in log_2_-space for determining the value of Φ(·). It has been suggested in separate works by Dudoit *et al*. [15] and Yang *et al*. [16] that a log_2_-based scatterplot of the average fluorescence intensity *A *versus the transformed ratio *M *should be used instead of a simple, log_2_-based intensity scatterplot. This type of scatterplot is commonly known as a Bland-Altman plot in the statistics literature. The values for *A *and *M *are given as,


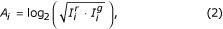



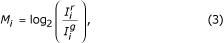


for *i *= 1, 2,..., *n*. Equations (2) and (3) are preferred over the original intensities because the (*A*, *M*)-scatterplot may reveal artifacts that are not clearly visible in the ordinary intensity scatterplot. Such a transformation represents a scaled, 45° rotation of the (*I*^*g*^, *I*^*r*^)-coordinate system [16].

The smoothing procedure has been designed to accommodate measured scatterplot data obeying the form *M*_*j *_= *g*(*A*_*j*_) + *ε*_*j*_, where the *j*th transformed ratio *M*_*j *_is a function of the corresponding overall intensity *A*_*j *_and a zero mean random variable *ε*_*j*_. The smoothed point at *A*_*j *_using LOWESS with a degree *d *polynomial is (*A*_*j*_, 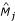), where 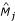 is the fitted value of the regression. The LOWESS estimate, 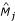, is a weighted linear combination of the *M*_*i*_


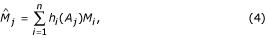


where the *h*_*i*_(*A*_*j*_) depend on *A*_*i*_, ∀*i*, but not on the *M*_*i*_. The LOWESS algorithm contains four data-specific parameters, namely the polynomial order *d*, the number of LOWESS algorithmic iterations *t*, the weight function *w*(·), and the fraction of the data points used in the local regression *f*. Consequently, these parameters all affect the values of the weights *h*_*i*_(*A*_*j*_) in Eq. (4). For a complete outline of the LOWESS algorithm, consult [8,14,27]. In practice, the polynomial order for DNA microarray data is usually selected as being either *d *= 0, 1, or 2, depending on the choice of (*I*^*g*^, *I*^*r*^)- or (*A*, *M*)-coordinate systems, the tri-cube weight function is quite standardized for all types of data [8], and the number of iterations is usually fixed at *t *= 3. The final parameter must be chosen where *f *∈ (0, 1] and it is often times assigned an arbitrary value without any justification. However, since the choice of *f *ultimately determines the magnitude of calibration, it is essential to put heavy emphasis on choosing this parameter carefully. In the literature, many microarray studies neglect such concerns and arbitrarily select *f *for different experimental data sets [12,13,16]. Formal consideration of the parameter *f *is typically glossed over by simply stating that the larger the *f *value, the smoother the fit. Although this is a true statement, the consequences are deeper than the statement leads on. Different types of data may require smoother fits but DNA microarray data takes all shapes. Also, what defines a *smoother *fit is also highly subject to interpretation depending on the actual data.

### The optimized approach

For a microarray experiment, there are a total of ℓ print-tips used on a single slide. In order to reliably determine the value of *f *for each print-tip group, we introduce an optimization approach based on the actual microarray data for each print-tip group. We slightly modify our notation to include print-tip indices as a subscript *k *for each transformed ratio. The goal is to select the appropriate values of *f*_*k *_that minimizes the mean squared difference between the LOWESS fit of the *i*th transformed ratio in the *k*th print-tip group, 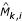, and the corresponding normalization reference level, *ψ*_*k*,*i*_(·). The value of each *ψ*_*k*,*i*_(·) is a function of experiment-specific parameters such as temperature or other environment settings which may differ from sample to sample in a single experiment. Accordingly, the cost function to be minimized for the *k*th print-tip group across all transformed ratios is


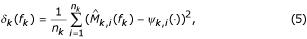


with the constraint that *f*_*k *_∈ (0, 1]. Here, the value *n*_*k *_is the total number of ratios for the *k*th print-tip group. Correspondingly, for a total of ℓ print-tip groups, we have 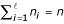. For certain experiments, like self versus self hybridizations, the true expression value is known *a priori*. If *ψ*_*k*,*i*_(·) is unknown, reliable estimates that reflect experiment-specific assumptions may be used. Usually there are tens of thousands of genes in a microarray study and a plausible assumption is that the mean of the log_2_-transformed ratios after normalization is zero. Also, in a variety of experiments, platform-dependent control transcripts that are known to have certain expression at a constant level may be utilized in the optimized approach. Furthermore, in our breast cancer case study we show how to obtain statistically reliable estimates of *ψ*_*k*,*i*_(·) from replicate slides. We also show how our approach may be used if replicates are not available for typical microarray studies. Ultimately, the optimized approach requires experimenters to explicitly state their assumptions behind the study, which is systematically better than arbitrarily choosing parameter values. In addition, determining an experiment-specific *f*_*k *_by trial and error may be time consuming and will oftentimes lead to non-optimal results. The chosen optimization algorithm for minimizing the corresponding cost function is based on a combination of golden-section search and successive parabolic interpolation as outlined by Forsythe *et al*. [28]. This approach finds the best *f*_*k *_for minimizing *δ*_*k*_(*f*_*k*_) for each print-tip, *k *= 1,..., ℓ within a tolerance of ±0.01. Each print-tip, resultingly, may have a different, optimal bandwidth parameter.

### Normalization step

After the estimates 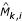 have been obtained, calibrating the intensities for all the *A*_*k*,*i *_is given as


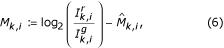


for *i *= 1,..., *n*_*k*_, and *k *= 1,..., ℓ. For the local LOWESS normalization within each print-tip group, the issue of how the total intensities are spread about the sample mean for the group becomes a factor to consider when normalizing the data [16]. After normalization, all the log_2_-ratios from the different print-tip groups are usually centered around zero. Some print-tips may have larger variances compared to others and an appropriate scale adjustment is needed to account for such differences. One proposed approach is to find the maximum likelihood estimate for the scale of the variance for each print-tip group [16]. This method assumes that all log_2_-ratios from the *k*th print-tip group follow a normal distribution with mean zero and variance 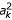
*σ*^2^, where *σ*^2 ^is the variance of the true log_2_-ratios and 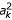 is the estimated scale factor for the *k*th print-tip group. However, this is only valid for certain types of data that reasonably follow a normal distribution and in our work we observe that this assumption may often times lead to undesirable results. Refer to [16] for further details.

Another approach proposed here that is able to deal with the variance scaling issue is to introduce a weighting factor in the calibration function that is of the form


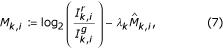


for *i *= 1,..., *n*_*k*_, *k *= 1,..., ℓ, and where the weight is given as 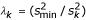. The bias-corrected sample variance for the *k*th print-tip is denoted by 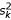 and is given as


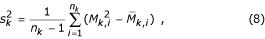


where 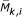 denotes the sample mean for print-tip *k*. Furthermore, the minimum sample variance is given as


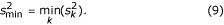


Compared to the maximum likelihood method outlined by [16], this method stresses higher weighting on print-tip groups that exhibit less variance and lower weighting for highly variant print-tips. If such a weight is not introduced, the normalization may improperly calibrate highly variant print-tip groups that have extreme sample means and many genes may erroneously be considered as differentially expressed as a consequence. Other treatments, such as the one suggested by Quackenbush [12] examine the geometric mean of the tip variances as a scale factor for the normalization estimate. However, such a treatment may not always scale the tips properly since some tips may still be overly compensated. Our proposed scaling factor *λ*_*k *_takes values over (0, 1] while other scaling methods may have larger upper limits. By calibrating data using Eq. (9), we have obtained nearly identical sample means, but less total variance for the resulting data compared to previously published techniques. The computation of *λ*_*k *_is straightforward and easy to calculate but our novel variance stabilization procedure does not take into account any heteroscedasticity in the data, namely observed increasing ratio variance with decreasing measurement intensity *A*. A rigorous comparison of print-tip scaling is beyond the scope of this contribution, but it is noted that the different scaling procedures affect the overall calibration scheme.

### Case studies

To demonstrate the utility of our optimized LOWESS normalization procedure, we first utilized a set of three self versus self experiments [21], BT-474, MCF-7, and HBL-100, which were obtained using the protocols delineated in the methods section. In addition, we calibrated a set of four breast cancer cell lines [22], BT-474, MCF-7, MDA-MB-436, and MDA-MB-361, each measured in comparison to the reference cell line HBL-100, which were obtained using the protocols outlined by Järvinen *et al*. [20]. For each cancer cell line, three replicate slide hybridizations were available. In order to reduce the effects of spots whose intensities are not reliable due to experimental or printing errors, we used two separate quality filtering methods and normalized the intensities after discarding values that were detected unreliable. The assessment of ratio quality was performed using the method proposed by Chen *et al*. [29] and the evaluation of spot quality was performed using the method of Hautaniemi *et al*. [30]. Optimized parameter selection for *f*_*k *_was performed and print-tip LOWESS normalization results are compared to the results using arbitrary choices of the parameter *f*_*k*_. The implementation took a few minutes to run on a standard desktop PC running MATLAB.

#### Self versus self experiments

**Figure 1:**
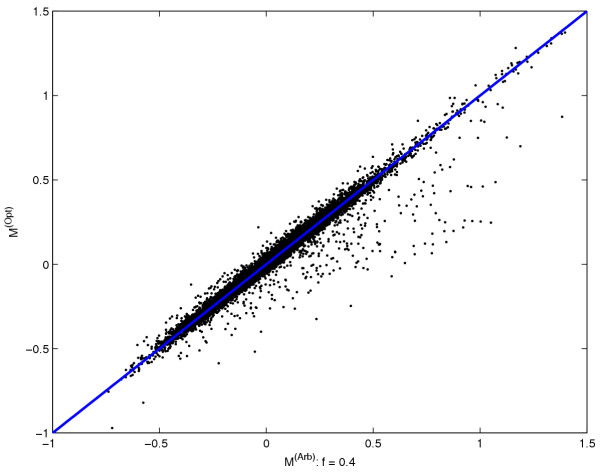
**(*M*^(Arb)^, *M*^(Opt)^)-Scatterplot analysis of BT-474 self versus self data **This plot compares the calibrated ratios obtained by LOWESS (*d *= 1) with the arbitrary choice of *f*_*k *_= 0.4 for all print-tips compared to ratios obtained with optimized *f*_*k *_for each print-tip group. The line of unity slope that passes through the origin shows where all the points should lay if both calibration methods produced identical ratios. For this self versus self experiment, the group of points that lay under this line shows that the arbitrary *f*_*k *_may improperly under-normalize these points.

Self versus self experiments provide a trivial application to test our method since the amount of mRNA in both the test and the reference samples is the same. Thus, the points of an intensity scatterplot in the log_2 _- log_2 _space should be distributed along a straight line that intersects zero with a slope of unity. In the (*A*, *M*)-coordinate system, all values of *M *should lie on a straight line at *M *= 0 for all values of *A*; this means that the calibrated ratios should ideally be unity for all variables. Correspondingly, the cost measure is given when *ψ*_*k*,*i*_(·) = 0, (∀*k*, *i*), in Eq. (5) for the (*A*, *M*)-coordinate systems. Separate trials were conducted using weighted, zeroth-order (*d *= 0), first-order (*d *= 1), and quadratic (*d *= 2) polynomial fits. For all trials, the number of print-tip LOWESS iterations was fixed at *t *= 3. The weight function used is given by Cleveland [8]. For each experiment, the local print-tip groups were separately normalized with their respective, optimized values of *f*_*k*_. As a comparison to arbitrary selections of *f*_*k*_, the print-tip normalization was also carried out using *f*_*k *_= 0.2, 0.4, 0.6, and 0.8 in separate trials. Figure 1 shows the (*M*^(Arb)^, *M*^(Opt)^)-scatterplot comparison between the calibration results with *d *= 1 using optimal *f*_*k *_and arbitrary *f*_*k *_for the BT-474 self versus self experiment. The points that deviate from the blue line are the genes that are most affected by the choice of *f*_*k*_. The *M*^(*Arb*) ^data in this figure was calibrated using *f*_*k *_= 0.4, ∀*k*.

In all three self versus self experiments, the global sample means of *M *were nearly the same after calibration, regardless of the choice of *f*_*k*_. However, the calibrations that used optimized selections of *f*_*k *_for each print-tip resulted in data that contained less overall variance compared to the arbitrary selections. The ultimate goal of calibration is to adjust the dynamic range for the transformed ratios and reduce the variability within the data. By using optimized selection of *f*_*k*_, we outperform all arbitrary formulations to achieve these goals.

#### Typical microarray experiments

One immediate concern for typical experimental microarray data is that many genes may be over- or under-expressed and the true, transformed gene expression ratio *ψ*_*k*,*i*_(·) surely will not be equal to zero for all genes. Accordingly, implementing the cost function in Eq. (5) becomes an immediate challenge since the normalization reference level of all the genes for a typical microarray experiment may be diffcult to determine with complete accuracy. We note that our cost function still may be used with the assumption that the sample mean for each print tip before log_2_-transformation is unity. In most microarray experiments, many genes may be assumed to have constant RNA concentrations while smaller numbers of genes may be over or under expressed, namely their sample mean over all the genes is zero, 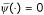. Using this assumption in Eq. (5), our experiments show that by minimizing the cost function in this context, like in the self versus self case study, we are able to systematically choose *f*_*k *_and the only consequence is that the minimum of the cost will not be as low as in the self versus self scenario where all genes should be constantly expressed. The main benefit of utilizing LOWESS for microarray normalization is that it is robust to extreme outliers and the cost function implemented in this fashion further restricts the effects of such extreme points in the regression. Ultimately, this implementation results in reliably calibrated ratios compared to the arbitrary formulation where different choices of *f*_*k *_affect the resulting data.

Since a single microarray experiment represents an observation, multiple observations would be needed to compute a reliable estimate of the true transformed ratio values. The use of only a small number of replicate slides may be satisfactorily used to determine reliable estimates of true gene expression and one study showed that three replicates suffce for significantly reducing experimental variability [31]. With the growing number of publicly available microarray data, conducting replicate experiments is becoming a popular solution to assess experimental errors and reduce noise bias in the measurements [32]. The advantages of replicate slides also greatly help the analysis of between-slide variability and help address formal statistical considerations when drawing biological conclusions. Here, we show that the optimized normalization approach may be directly extended in an iterative manner to use the estimates of the true ratio values for further specifying *f*_*k*_. After an initial round of optimized LOWESS normalization for each replicate slide with *ψ*_*k*,*i*_(·) = 0 in Eq. (5), the sample mean for each gene may then be calculated using the replicates. The normalization reference levels *ψ*_*k*,*i*_(·) were reassigned these average gene expression values in Eq. (5). Each experiment was then separately calibrated a second and final time using the optimization approach and the final results were noticeably different compared to the normalized data using *f *= 0.2 that Järvinen *et al*. posted on their website [20]. A noteworthy consideration to address here is the overall effect of an iterative calibration process on the underlying structure of the data. Experimentally, once the optimized LOWESS regression is computed using the average value for each gene and normalization is performed, subsequent calibration attempts using the cost function-based method do not result in drastically different data. The subsequent regressions are nearly constant lines near *M *= 0 in the (*A*, *M*)-scatterplot if the cost function approach is used. Consequently, the calibrated data reach a stable domain with a small dynamic range. Empirically, we found that performing optimized normalization in an iterative manner will not propagate regression effects through to disrupt the underlying structure of the data.

**Figure 2:**
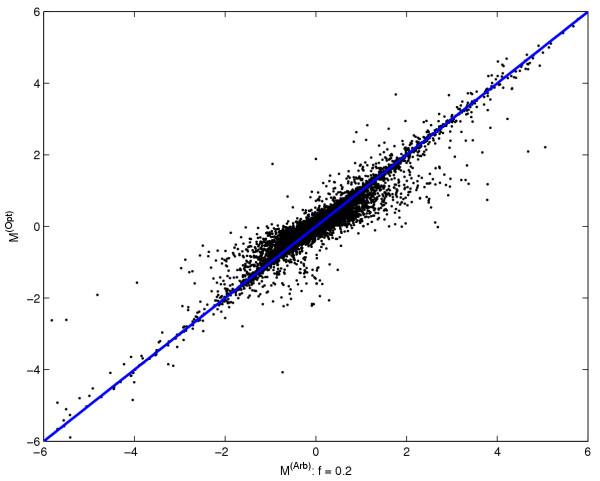
**(*M*^(Arb)^, *M*^(Opt)^)-Scatterplot analysis of BT-474_01 data **This plot compares the calibrated ratios obtained by LOWESS (*d *= 1) with arbitrary (*f*_*k *_= 0.2) and optimized bandwidth windows for the first replicate hybridization of the BT-474 breast cancer cell line. Again, the line of unity slope shows where all the points should lay if both calibration methods produced identically calibrated ratios. Many points deviate from the similarity line in this example and such results are commonly observed for the microarray data used in this study. Consequently, it is clear that the choice of *f*_*k *_greatly affects how the data is calibrated. Points that are furthest away from the similarity line are highly influenced by the choice of *f*_*k *_in LOWESS calibration.

**Figure 3:**
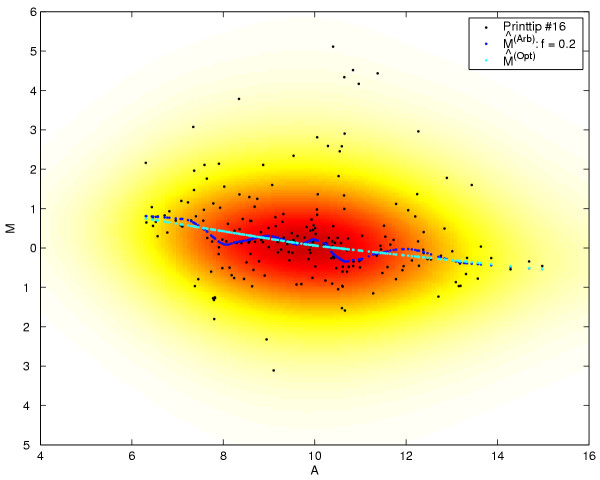
**Print-tip LOWESS comparisons for BT-474_01 data **This (*A*, *M*)-scatterplot shows a two-dimensional histogram [33] or all the spots for the first replicate BT-474 breast cancer hybridization, where the bright red color indicates a high concentration of spots. Print-tip *k *= 16 is highlighted by black dots. The LOWESS estimates obtained by using *f*_16 _= 0.2 are shown by the dark blue line and the estimates using optimal *f*_16 _is shown here in light blue. This result is typical for the print-tips in this study based on minimizing the cost function given in Eq. (5).

**Figure 4:**
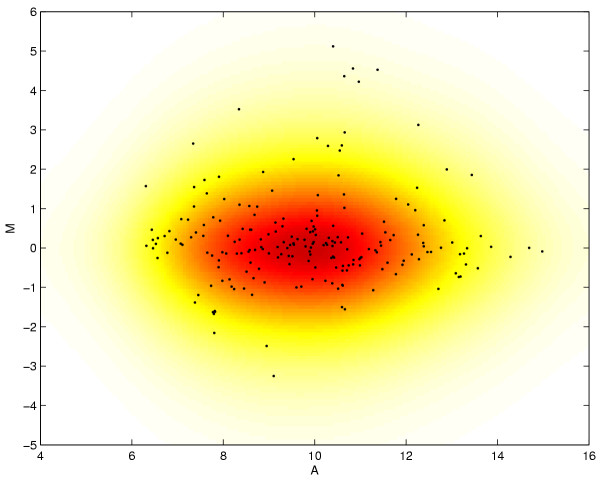
**Arbitrary calibration results for BT-474_01 data **All spots are shown using a two-dimensional scatterplot with the spots from print-tip *k *= 16 are highlighted here in black. LOWESS calibration has been performed using the choice of *f*_*k *_= 0.2 for all print-tips.

**Figure 5:**
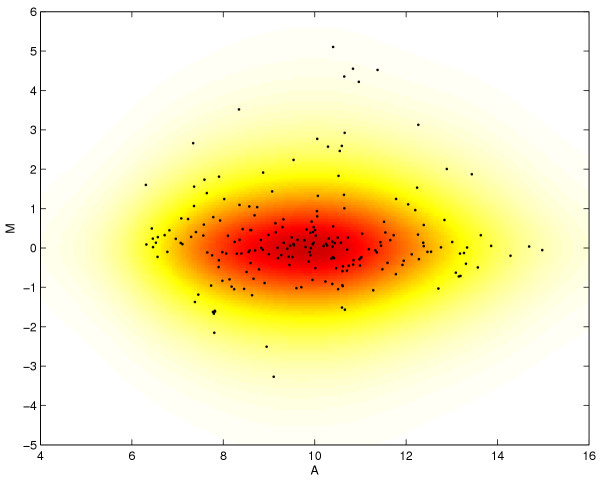
**Optimized calibration results for BT-474_01 data **This scatterplot shows LOWESS calibration after optimized choices of *f*_*k *_have been obtained for all print-tips. Compared to the results in Figure 4, the normalized data here has less overall variance. In addition, genes that have been verified experimentally conform in better agreement with the well-known biology.

Figure 2 shows the scatterplot comparison between the calibration results using optimal and arbitrary selections of *f*_*k *_for the first replicate BT-474 hybridization. Some genes in this plot report 4-fold differences and ultimately these differences affect data analysis. Consequently, the errant choice of this parameter *f*_*k *_may have deleterious effects on different biological studies. To illustrate the differences for one representative print-tip in this breast cancer study for the first replicate of the BT-474 cell line, Figure 3 plots the regressions obtained by both methods. All the data points for this hybridization are shown as a two-dimensional histogram [33], while the spots given by print-tip *k *= 16 are highlighted in black. In this plot, we show that the regression obtained by the optimized choice of *f*_16 _differs from the one obtained by arbitrarily selecting *f*_16 _= 0.2 and the calibration results are thus affected. Figure 4 reports arbitrary calibration results and Figure 5 shows optimized results. The data in Figure 5 has less overall variance when calibrated with the optimized choices of *f*_*k*_.

As further illustration of the calibration differences between the optimized and arbitrary calibration results, we employ a goodness-of-fit test [34]. We wish to make a direct test of the data, independent of any underlying parent distribution of the ratios, and we use the following statistic for the *k*th print-tip group


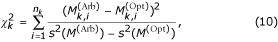


where *M*^(Arb) ^and *M*^(Opt) ^are the arbitrary and optimized calibration results, and the denominator within the summation is simply the variance of the difference between *M*^(Arb) ^and *M*^(Opt)^. The null hypothesis is defined to be *H*_0_: the normalized ratios using arbitrary *f *are comparable to ones using optimized *f*. We tested against *p *< 0.05 for the 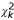 distribution and reported the alternative hypothesis for a few print-tip groups on almost all the slides. In this analysis, we compared optimized choices of *f *for each print-tip to the arbitrary choices *f *= 0.2, 0.4, 0.6, and 0.8. By looking across each replicate of the calibrated data for all four breast cancer cell lines, almost all slides in this study reported at least one print-tip to have statistically different calibration results based on the choice of *f*_*k*_. Often times a single slide would report two or three print-tip groups that had statistically different calibration results.

In addition to statistical analysis, genes that exhibit known over-expression in the BT-474 cell line data [35] were selected here for more detailed analysis. In particular, genes that were verified experimentally using reverse transcription-polymerase chain reaction (RT-PCR) were of the highest interest. Comparing our optimized calibration results utilizing the replicate data to the normalized data by Järvinen *et al*. [20], our results conform strongly with most of the over-expressed genes given in a list from a parallel study [35]. Two genes in particular stand out to demonstrate the benefits of utilizing our proposed method: *homeo box B7*, which was validated with RT-PCR [35], and *v-erb-b2*, which is known to be over-expressed in the BT-474 cell line [35]. The results posted by Järvinen *et al*. [20] for calibrating the *homeo box B7 *gene shows that it falls within the top 18% of overall gene expression, but by using the optimized approach we report it to be within the top 13%. For the *v-erb-b2 *gene, both calibration techniques report that this gene falls within the top 1% of the genes in terms of expression. As a result, for the *homeo box B7 *gene, the calibration factor *f*_*k *_is responsible for about 5% change in the reported gene expression. This is a dramatic result that may influence how the expression for this gene may be interpreted in comparison to the accepted biological knowledge of a certain experiment. As public data from microarray experiments continues to become available, the knowledge of certain genes will undoubtedly be uncovered for well-studied cell lines and this information will help further assess normalization and microarray quality control tasks.

## Conclusions

The LOWESS method has recently been applied in other applications for the biological sciences. Comparative genomic hybridization (CGH) is a molecular cytogenetic method of screening a tumor for genetic changes. The alterations are classified as DNA gains and losses and they reveal a characteristic pattern that includes mutations at chromosomal and subchromosomal levels. Our proposed optimized scheme is directly applicable to the application of calibrating CGH microarray experiments, as well as for data analysis aspects. For example, the work of Clark *et al*. [36] utilized the LOWESS method for identifying the regions where gene copy numbers were aberrantly high or low in prostate cancer using CGH microarray technology. The parameter *f *was chosen arbitrarily and its value was not reported in the study. Consequently, reproduction and verification of these results may be diffcult. For instance, some of the important biological findings, such as start and end points of amplifications and deletions, may be adversely affected by different choices of *f*.

In addition to CGH analysis, LOWESS has found application in case-control studies where logistic regression has been used to model the relationship between binary responses and continuous predictor variables [37]. In these types of studies one may use LOWESS to remove systematic trends that contaminate the laboratory measurements of predictor variables. The analysis reported by Borkowf *et al*. [37] clearly shows that different choices of *f *result in noticeably different correction effects and the optimization method proposed here may be suitable for enhancing such a study. Adaptations to the cost function may be utilized to handle this type of data. In addition, analysis of other types of scatterplot data by utilizing the LOWESS method with an arbitrary choice for the bandwidth parameter is undoubtedly susceptible to varied interpretations or errant conclusions [38,39].

Another result of this optimized calibration study is that we uncovered a better understanding of choosing the parameter *d *in the weighted polynomial fit. A higher-order (*d *> 2), weighted polynomial is rarely needed based on the argument that such an assumption is, to a certain extent, over-fitting the data. From the findings of our study, we find that it is better to use a linear estimate based on minimizing the estimate errors across (*A*, *M*)-scatterplots. Consequently, different choices of *d *resulted in different optimized values for *f*. The reason is that for the higher-order polynomial, it is beneficial in general to retain a larger fraction of the values of *A *for the weight function in computing the polynomial coeffcients. It is very important to carefully select *f *since ultimately, the bandwidth is a function of the polynomial order.

Here, we also reaffirmed the idea that the quality filtering of ratios and spots is a necessary step that should precede all experimental microarray data handling procedures, whether it is scatterplot-based normalization or any other normalization method, since errant ratios would surely have a deleterious affect on the calibration. For instance, in the BT-474 data, the first replicate slide had poor ratio quality for a handful of genes. Calibration without considering or removing these errant spots resulted in less reliable results. This study addresses the issue of locating sources of experimental error for print-tips that have high sensitivity for the parameter *f *. For one, print-tips are physically different and they are considered to have different types of errors introduced based on these properties. In the formulation of normalization, it is imperative to address such subtle issues when choosing and implementing any algorithm.

The systematic choice of the parameters in the LOWESS algorithm has not been previously addressed in the microarray literature and the method proposed here may be utilized in different microarray platforms. Such a treatment is also important for a wide variety of applications that employ scatterplot-based regression. The findings of this study illustrate that by choosing different values of *f *for the LOWESS algorithm results in noticeably different normalization results. This proposed method requires the calibration step to clearly state the assumptions used for within-slide normalization. Our optimization algorithm is more systematic than simply choosing an arbitrary parameter value or through trial and error techniques since the optimized approach relies on the actual underlying structure of the data. We also stress that such an optimization algorithm may also be utilized for other studies in addition to DNA microarray normalization treatments. Proper changes need to be made to Eq. (5) to reflect the ideal model for the data captured in the function *ψ*_*k*,*i*_(·), but in some studies, such a function may be satisfactorily determined or estimated from the data.

## Methods

### Data resources

For the self versus self hybridizations, custom cDNA microarray experiments proceed as follows. Altogether, three microarray hybridizations were performed using custom printed cDNA microarray slides from the same print batch. Labelling, hybridization and washing were done as described previously by Monni *et al*. [40] and Järvinen *et al*. [20]. Briefly, total RNA was extracted from cell lines BT-474, HBL-100, and MCF-7 and labelled with Cy3-dUTP and Cy5-dUTP (Amersham Biosciences, Piscataway, NJ). The custom printed cDNA microarrays comprised of 11,520 clones from Incyte Genomics IRAL cDNA library and 1,136 clones from Research Genetics library. Microarrays were printed on poly-l-lysine coated slides using an Omnigrid arrayer (GeneMachines) as described previously [20]. Microarrays were scanned with an Agilent laser confocal scanner (Agilent Technologies, Palo Alto, CA) and gridded using the DEARRAY software developed by Chen *et al*. [29]. For the four breast cancer cell lines, custom cDNA microarray experiments were provided in a separate contribution by Järvinen *et al*. [20] and detailed protocols are described in that work. The relevant genes in our study were verified using RT-PCR in a parallel study by Hyman *et al*. [35].

### Data quality filtering

All microarray experiments contained in this work were conducted and spotted using groups of ℓ = 32 print-tips, with each tip being responsible for either 384 or 420 spots in their respective subarray. In order to reduce the effects of spots whose intensities are not reliable due to experimental or printing errors, we used two separate quality filtering methods and normalized the intensities after discarding values that were detected unreliable. The assessment of ratio quality was performed using the method proposed by Chen *et al*. [29] and ratios that had a quality value below the threshold 0.5 were discarded from our analysis. This quality cutoff value has, in the past, been shown to represent less reliable cDNA microarray measurements due to either low signal intensity, high local background level, uneven distribution of the target intensity, and/or small target size. The evaluation of spot quality was performed using the method of Hautaniemi *et al*. [30]. In this Bayesian networks-based method, we utilized the following features in determining spot quality. Bleeding, spot roundness, and spot intensity were assessed for the Cy5 channel and bleeding, spot size, spot roundness, background intensity, and fitting error were evaluated for the Cy3 channel. These features were chosen since this set was found to result in the best classification accuracies [30]. The trained Bayesian network was applied to each slide in this study and all the spots having a quality value of zero were excluded from the subsequent analysis.

## Authors' contributions

JAB developed the mathematical formulation of the problem, implemented the optimized normalization algorithm in MATLAB, developed the statistical analysis, and wrote the manuscript. SH developed the LOWESS normalization software in MATLAB, coordinated spot quality filtering, and assisted in drafting the manuscript. AKJ conducted the self versus self microarray experiments and performed ratio quality filtering for data analysis. HE assisted in data preparation and in drafting the manuscript. SKM participated in the design and coordination of the study and assisted in drafting the manuscript. JA reviewed the statistical analysis and participated in the design and coordination of the study. All authors read and approved the final manuscript.

## References

[B1] Schena M, Shalon D, Davis RW, Brown PO (1995). Quantitative monitoring of gene expression patterns with a complementary DNA microarray. Science.

[B2] Goryachev AB, MacGregor PF, Edwards AM (2001). Unfolding of microarray data. Journal of Computational Biology.

[B3] Ideker T, Thorsson V, Siegel AF, Hood LE (2000). Testing for differentially-expressed genes by maximum-likelihood analysis of microarray data. Journal of Computational Biology.

[B4] Kerr MK, Martin M, Churchill GA (2000). Analysis of variance for gene expression microarray data. Journal of Computational Biology.

[B5] Tseng GC, Oh MK, Rohlin L, Liao JC, Wong WH (2001). Issues in cDNA microarray analysis: quality filtering, channel normalization, models of variations and assessment of gene effects. Nucleic Acids Research.

[B6] Wang X, Ghosh S, Guo SW (2001). Quantitative quality control in microarray image processing and data acquisition. Nucleic Acids Research.

[B7] Dobbin K, Shih JH, Simon R (2003). Statisical design of reverse dye microarrays. Bioinformatics.

[B8] Cleveland WS (1979). Robust locally weighted regression and smoothing scatterplots. Journal of the American Statistical Association.

[B9] Ihaka R, Gentleman R (1996). R: a language for data analysis and graphics. Journal of Computational and Graphical Statistics.

[B10] Engelen K, Coessens B, Marchal K, Moor BD (2003). MARAN: normalizing micro-array data. Bioinformatics.

[B11] Venet D (2003). MatArray: a Matlab toolbox for microarray data. Bioinformatics.

[B12] Quackenbush J (2002). Microarray data normalization and transformation. Nature Genetics.

[B13] Yang YH, Dudoit S, Luu P, Lin DM, Peng V, Ngai J, Speed TP (2002). Normalization for cDNA microarray data: a robust composite method addressing single and multiple slide systematic variation. Nucleic Acids Research.

[B14] Cleveland WS, Devlin SJ (1988). Locally weighted regression: an approach to regression analysis by local fitting. Journal of the American Statistical Association.

[B15] Dudoit S, Yang YH, Callow MJ, Speed TP (2002). Statistical methods for identifying genes with differential expression in replicated cDNA microarray experiments. Statistical Sinica.

[B16] Yang YH, Dudoit S, Luu P, Speed TP, Bittner M, Chen Y, Dorsel A, Dougherty ER (2001). Normalization for cDNA microarray data. In Microarrays: optical technologies and informatics.

[B17] Bolstad BM, Irizarry RA, Åstrand M, Speed TP (2003). A comparison of normalization methods for high density oligonucleotide array data based on variance and bias. Bioinformatics.

[B18] Edwards D (2003). Non-linear normalization and background correction in one-channel cDNA microarray studies. Bioinformatics.

[B19] Wilson DL, Buckley MJ, Helliwell CA, Wilson IW (2003). New normalization methods for cDNA microarray data. Bioinformatics.

[B20] Järvinen AK, Hautaniemi S, Edgren H, Auvinen P, Saarela J, Kallioniemi OP, Monni O (2004). Are data from different gene expression microarray platforms comparable?. Genomics.

[B21] Supplementary Webpage (Self vs. Self data). http://www.ece.ucsb.edu/pubs/bmc/.

[B22] Supplementary Webpage (Breast Cancer data). http://sigwww.cs.tut.fi/TICSP/Jarvinen_et_al_2003/.

[B23] Dobbin K, Shih JH, Simon R (2003). Questions and answers on design of dual-label microarrays for identifying differentially expressed genes. J Nat Cancer Inst.

[B24] Duggan DJ, Bittner M, Chen Y, Meltzer P, Trent JM (1999). Expression profiling using cDNA microarrays. Nature Genetics.

[B25] Finkelstein D, Ewing R, Gollub J, Sterky F, Cherry JM, Somerville S (2002). Microarray data quality analysis: lessons from the AFGC project. Plant Molecular Biology.

[B26] Holloway AJ, van Laar RK, Tothill RW, Bowtell DDL (2002). Options available – from start to finish – for obtaining data from DNA microarrays II. Nature Genetics.

[B27] Fan J, Gijbels I (1996). Local Polynomial Modelling and its Applications.

[B28] Forsythe GE, Malcolm MA, Moler CB (1977). Computer Methods for Mathematical Computations.

[B29] Chen Y, Kamat V, Dougherty ER, Bittner ML, Meltzer PS, Trent JM (2002). Ratio statistics of gene expression levels and applications to microarray data analysis. Bioinformatics.

[B30] Hautaniemi S, Edgren H, Vesanen P, Wolf M, Järvinen AK, Yli-Harja O, Astola J, Kallioniemi O, Monni O (2003). A Novel Strategy for Microarray Quality Control Using Bayesian Networks. Bioinformatics.

[B31] Lee M, Kuo F, Whitmore G, Sklar J (2000). Importance of replication in microarray gene expression studies: statistical methods and evidence from repetitive cDNA hybridizations. Proc Natl Acad Sci USA.

[B32] Yang YH, Speed TP (2002). Design issues for cDNA microarray experiments. Nature Reviews Genetics.

[B33] Eilers PHC, Goeman JJ (2004). Enhancing scatterplots with smoothed densities. Bioinformatics.

[B34] Bevington PR, Robinson DK (1992). Data Reduction and Error Analysis for the Physical Sciences.

[B35] Hyman E, Kauraniemi P, Hautaniemi S, Wolf M, Mousses S, Rozenblum E, Ringnér M, Sauter G, Monni O, Elkahloun A, Kallioniemi OP, Kallioniemi A (2002). Impact of DNA amplification on gene expression patterns in breast cancer. Cancer Research.

[B36] Clark J, Edwards S, Feber A, Flohr P, John M, Giddings I, Crossland S, Stratton MR, Wooster R, Campbell C, Cooper CS (2003). Genome-wide screening for complete genetic loss in prostate cancer by comparative hybridization onto cDNA microarrays. Oncogene.

[B37] Borkowf CB, Albert PS, Abnet CC (2003). Using lowess to remove systematic trends over time in predictor variables prior to logistic regression with quantile categories. Statistics in Medicine.

[B38] Mazerolle M (2003). Detrimental effects of peat mining on amphibian abundance and species richness in bogs. Biological Conservation.

[B39] Hen I, Sakov A, Kafkafi N, Golani I, Benjamini Y (2004). The dynamics of spatial behavior: how can robust smoothing techniques help?. Journal of Neuroscience Methods.

[B40] Monni O, Bärlund M, Mousses S, Kononen J, Sauter G, Heiskanen M, Paavola P, Avela K, Chen Y, Bittner M, Kallioniemi A (2001). Comprehensive copy number and gene expression profiling of the 17q23 amplicon in human breast cancer. Proc Natl Acad Sci.

